# A long-term longitudinal study of the osteoarthritic changes to the temporomandibular joint evaluated using a novel three-dimensional superimposition method

**DOI:** 10.1038/s41598-021-88940-y

**Published:** 2021-04-30

**Authors:** Kyungjae Han, Mun Cheol Kim, Youn Joong Kim, Yunheon Song, Ilho Tae, Jae-Jun Ryu, Dong-Yul Lee, Seok-Ki Jung

**Affiliations:** 1 Department of Orthodontics, Korea University Guro Hospital, Seoul, 08308 Republic of Korea; 2 Department of Orthodontics, Graduate School of Clinical Dentistry, Korea University, Seoul, 02841 Republic of Korea; 3 TMJ and Orofacial Pain Center, Ahrim Dental Hospital, Seoul, 06169 Republic of Korea; 4 Department of Prosthodontics, Korea University Anam Hospital, Seoul, 02841 Republic of Korea; 5 Department of Orthodontics, Korea University Ansan Hospital, 123 Jeokgeum-ro, Danwon-gu, Ansan-si, Gyeonggi-do 15355 Republic of Korea

**Keywords:** Osteoarthritis, 3-D reconstruction, X-ray tomography

## Abstract

The aim of this study was to assess the changes in individual condyles from 5 to 8 years in patients with temporomandibular joint (TMJ) osteoarthritis using 3-dimensional cone beam computed tomography (3D CBCT) reconstruction and superimposition. To assess the longitudinal TMJ changes, CBCT was performed at initial (T_0_) and final (T_2_) timepoints that were at least 5 years apart and at a middle (T_1_) timepoint. To improve the accuracy, we used a novel superimposition method that designated areas of coronoid process and mandibular body. The differences in the resorption and apposition amounts were calculated between each model via maximum surface distances. The greatest resorption and apposition observed were − 7.48 and 2.66 mm, respectively. Evaluation of the changes in each condyle showed that osteoarthritis leads to both resorption and apposition. Resorption was mainly observed in the superior region, while high apposition rates were observed (in decreasing order) in the posterior, lateral, and anterior regions. The medial parts showed greater apposition than the lateral parts in all regions. Our superimposition method reveals that both resorption and apposition were observed in condyles with TMJ osteoarthritis, and resorption/apposition patterns depend on the individual condyle and its sites.

## Introduction

The temporomandibular joint (TMJ) is a complex structure that is affected by high load activities and parafunctional oral habits. When a force exceeding the joint's capacity is applied, degenerative changes occur with secondary inflammatory changes in the tissues and hard tissue destruction^1–3^. Changes associated with TMJ osteoarthritis vary from disc displacement to subchondral bone alteration (erosion), bone overgrowth (osteophytes), and fibrocartilage loss^4–6^. In addition, TMJ osteoarthritis can be diagnosed only when the following changes are observed accurately. Thus, various imaging methods have been developed for this purpose.

With the development of cone beam computed tomography (CBCT), tissue changes caused by TMJ osteoarthritis can be observed better. Osteoarthritic bone changes in the hard tissue, such as erosion, flattening, and osteophytes, can be observed using CBCT, which also allows for observation of condylar tissue absorption or deposition^7,8^. Studies have reported that condylar resorption was followed by healing and apposition in TMJ osteoarthritis. Specifically, studies have shown that apposition occurs when splint therapy is performed^8–11^. However, changes of TMJ occur in three dimensions. There are complex changes on each direction. Therefore, it is more accurate to see the change of TMJ as a three-dimensional vector more than 2-dimensional (2D) image to see the actual change of the condyle.

To overcome CBCT’s limitation where only a 2D layer can be observed, CBCT scans were reconstructed with a 3-dimensional (3D) view for better TMJ visualization^9^. By reconstructing the CBCT scans’ 2D cross-sectional images into 3D images, localization and quantification of condylar resorption patterns, which could not be assessed previously, became possible^12^. This method not only allowed for observation of the morphological changes seen in patients with TMJ osteoarthritis, but also of the resorption and apposition tendencies of the condyle and its specific sites^13–16^.

In the 3D studies, condylar changes were observed by overlaying images based on the condylar head or by creating an average model from several condyles. However, the condylar head-based superimposition method was only feasible when there were few condylar changes. If the follow-up period exceeded 5 years and the condyle underwent significant osteoarthritic changes, condylar head-based superimposition led to significant errors. Moreover, the limitation of studies that used an average condylar model was that each condyle differs in its pattern of change and size for each individual with osteoarthritis. Therefore, a new superimposition method was needed to observe osteoarthritic condylar changes^17–19^.

Additionally, although condylar changes caused by TMJ osteoarthritis are longstanding, there are few studies on the long-term changes. Most studies were performed over approximately 1 year and only changes at initial and final timepoints were assessed^13,20^. Osteoarthritic condylar changes alternate between resorption and apposition, and these patterns differ in each of the anterior, superior, posterior, and lateral condylar regions. Therefore, a longitudinal assessment of TMJ osteoarthritis was necessary to observe condylar changes.

Here, condylar changes were assessed at 3 timepoints: initial (T_0_), middle (T_1_), and final (T_2_) for a period in the range of 5–8 years to observe the condylar changes over time caused by long-term TMJ osteoarthritis. Moreover, to observe the significant changes that the condyles underwent over the 5 years, 3D superimposition, with improved accuracy, was used for the mandibular ramus and coronoid process. Thus, the aim of this study was to assess individual condylar changes for 5–8 years in patients with TMJ osteoarthritis using a 3D CBCT superimposition technique.

## Results

### Resorption and apposition amounts

**Figure 1:**
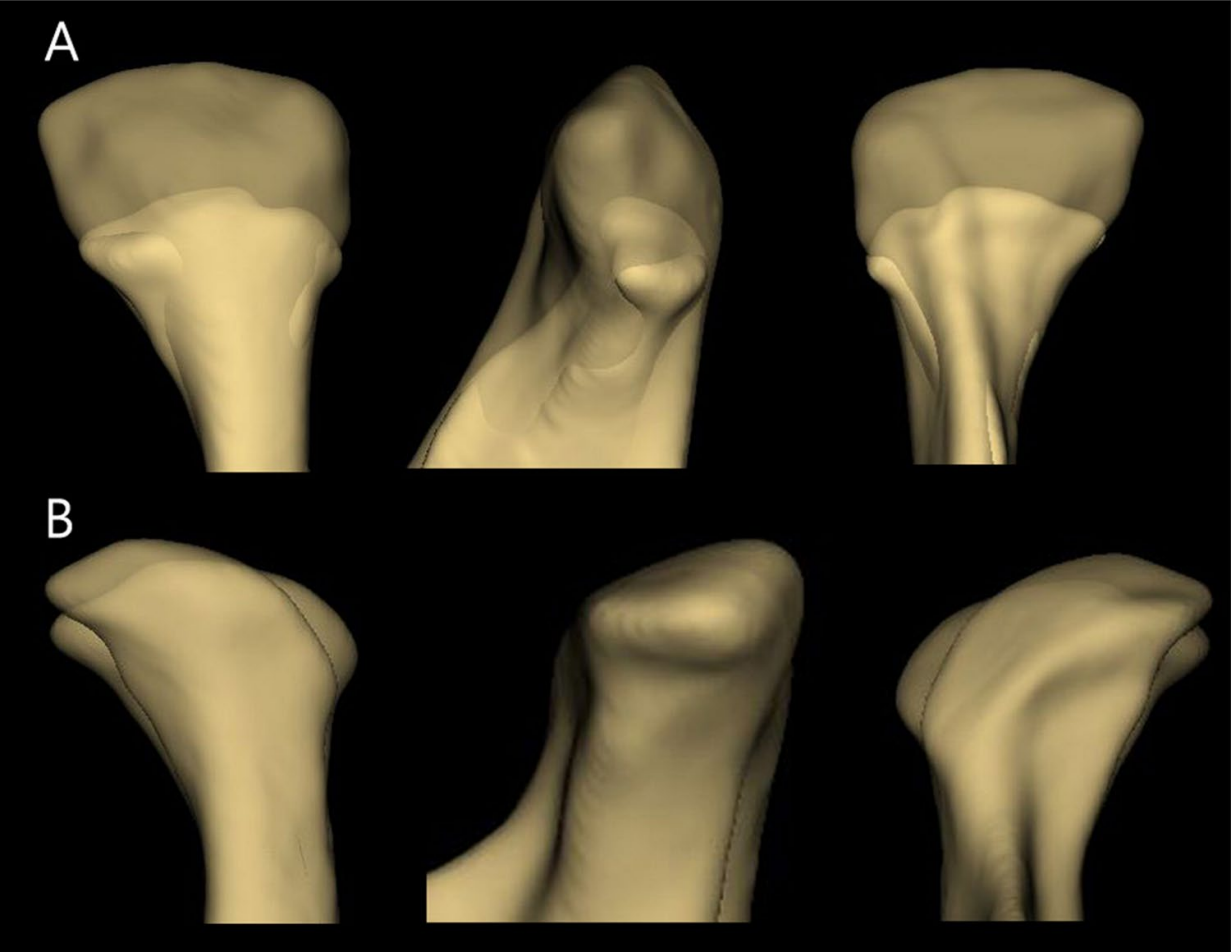
(**A**) Mandibular condyle with maximum resorption. (**B**) Mandibular condyle with maximum apposition, 3D slicer (v. 4.11.0, Slicer, http://www.slicer.org).

The greatest amount of resorption (− 7.48 mm) was observed in the superior condylar region. In 2 out of 67 condyles, resorption of − 7.48 mm was observed, and the follow-up periods were 5 years 10 months, and 4 years 7 months (Fig. 1A). The highest apposition (2.66 mm) was also observed in the superior region, and the follow-up period was 5 years and 3 months. However, in the models where the maximum apposition was observed, the medial part of the superior region underwent apposition while resorption was simultaneously observed in the lateral part of the superior region (Fig. 1B). The mean resorption and apposition amounts during the average follow-up period of 5 years and 6 months were − 2.69 mm and 1.44 mm, respectively. In addition, − 0.5 mm and 0.26 mm of resorption and apposition were observed per year, respectively. The maximum and average amounts, and changes per 1 year of resorption were greater than those of apposition (Table 1).

**Table 1:** Quantitative assessment of condylar surface changes using point-to-point distances on the registered condylar models between T_0_ and T_2_.

	Resorption (mm)	Apposition (mm)	
Max	− 7.48	2.66	
Average (SD)	− 2.69 (1.97)	1.44 (0.50)	
Per year (SD)	− 0.50 (0.38)	0.26 (0.11)	

Resorption	Male	Female	*p* value
Max	− 7.34	− 7.48	0.192
Average (SD)	− 2.34 (1.65)	− 2.79 (2.04)	
Per year (SD)	− 0.44 (0.34)	− 0.52 (0.39)	

Values are expressed as mean (standard deviation). *SD* standard deviation.

We compared the resorption amounts in 15 male and 54 female models. There were no significant differences in the resorption between the groups (Table 1).

### Changes in resorption and apposition of the superior region

The effect of the superior condylar region on the mandibular angle was dependent on resorption and apposition. Thus, they could cause changes in the skeletal pattern of the face. During a 5-year follow-up period, resorption and apposition of the superior condylar region were assessed from T_0_ to T_1_ and T_1_ to T_2_.

**Figure 2:**
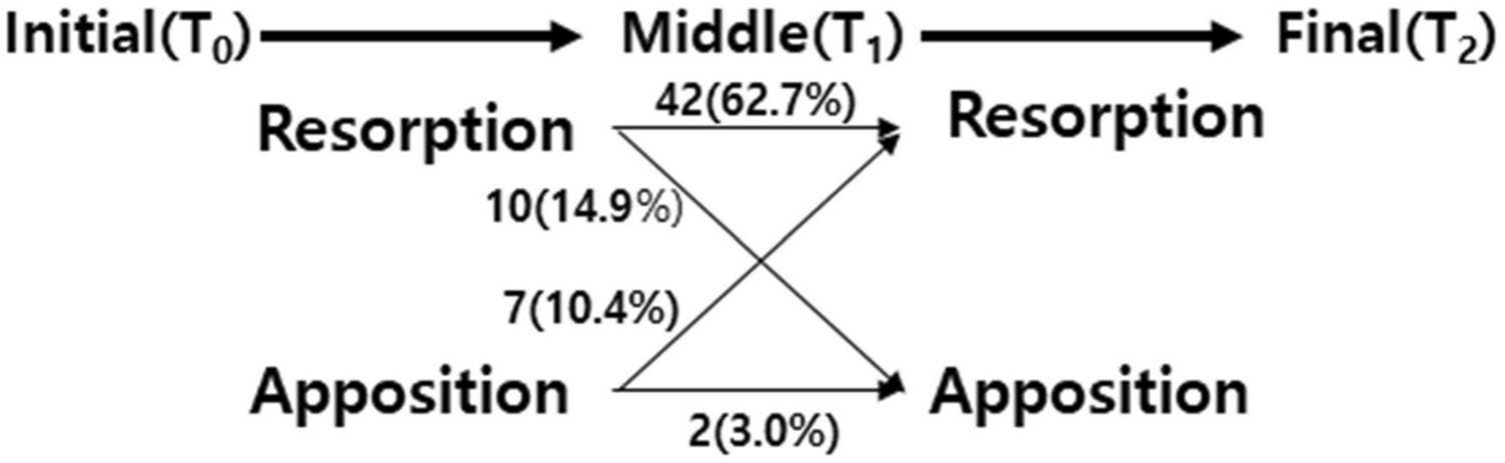
Changes in resorption and apposition of the superior part of condyle over time. T_0_, initial timepoint; T_1_, middle timepoint; T_2_, final timepoint.

Resorption was observed in 52 and 48 out of 67 condyles from T_0_ to T_1_ and T_1_ to T_2_, respectively. There were differences in the resorption and apposition case numbers between the periods of T_0_–T_1_ and T_1_–T_2_, as some condyles underwent resorption followed by apposition, and vice versa. Changes in resorption and apposition in the superior part from T_0_ to T_1_ and T_2_ are shown in Fig. 2.

**Figure 3:**
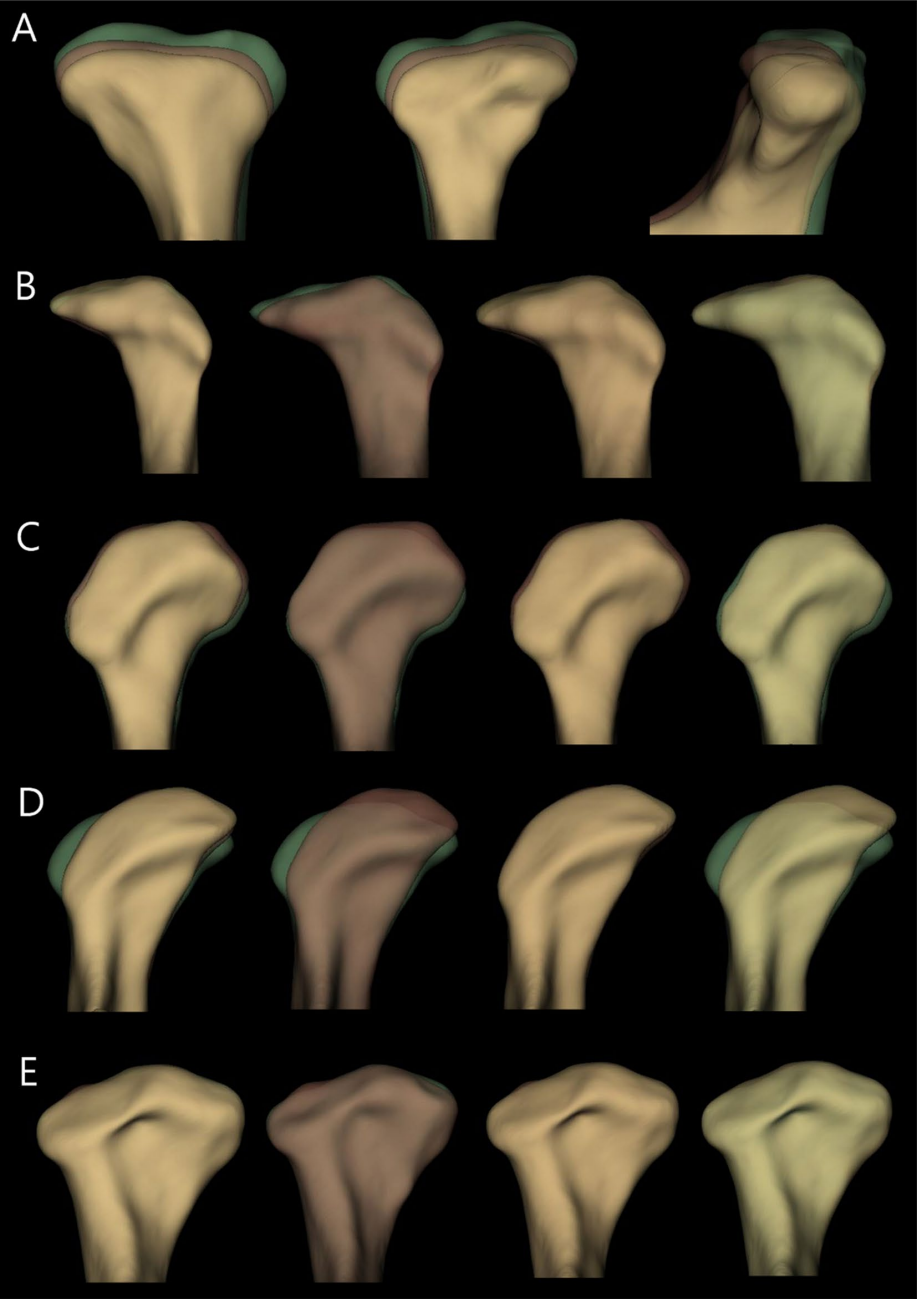
Mandibular condyles showing resorption and apposition changes in the superior part (**A**) Resorption → Resorption (**B**) Resorption → Apposition (**C**) Apposition → Resorption (**D**) Apposition → Apposition (**E**) No change. Green: T_0_, red: T_1_, and yellow: T_2_. T_0_, initial timepoint; T_1_, middle timepoint; T_2_, final timepoint, 3D slicer (v. 4.11.0, Slicer, http://www.slicer.org).

We found that 42, 10, 7, and 2 (62.7%, 14.9%, 10.4%, and 3.0%, respectively) condyles underwent resorption followed by resorption, resorption followed by apposition, apposition followed by resorption, and apposition followed by apposition, respectively, from T_0_ to T_1_ to T_2_. The corresponding condyles for each resorption and apposition pattern are shown in Fig. 3.

### Resorption and apposition sites

As changes from T_0_ to T_1_ and T_2_ were assessed, resorption was mainly observed in the superior region. In the lateral part of the superior region, resorption was observed at the same or higher frequency than the medial part at all timepoints; however, the differences were not significant. Although it was also statistically insignificant, resorption occurred more frequently in the lateral than in the medial part of the lateral region. Additionally, resorption was more frequent in the anterior than in the posterior region (Table 2).

**Table 2:** Condylar remodeling type frequencies in each section.

	Anterior (%)		Superior (%)		Posterior (%)		Lateral (%)		Total
	AM (%)	AL (%)	SM (%)	SL (%)	PM (%)	PL (%)	M (%)	L (%)	
**Resorption**									
T_0_–T_1_	45 (23.6)		86 (45.0)		28 (14.7)		32 (16.8)		
	25 (13.1)	20 (10.5)	42 (22.0)	44 (23.0)	12 (6.3)	16 (8.4)	15 (7.9)	17 (8.9)	191
*p* value	0.549		0.774		0.289		0.815		
T_1_–T_2_	46 (27)		74 (37.0)		36 (18.0)		52 (26.0)		
	20 (10.0)	18 (9.0)	37 (18.5)	37 (18.5)	16 (8.0)	20 (10.0)	26 (13.0)	26 (13.0)	200
*p* value	0.815		1		0.424		1		
T_0_–T_2_	48 (21.7)		97 (43.7)		31 (14.0)		46 (20.8)		
	27 (12.2)	21 (9.5)	47 (21.2)	50 (22.5)	11 (5.0)	20 (9.0)	21 (9.5)	25 (11.3)	222
*p* value	0.238		0.508		0.022		0.541		
**Apposition**									
T_0_–T_1_	41 (25.8)		21 (13.2)		50 (31.5)		47 (29.6)		
	24 (15.1)	17 (10.7)	12 (7.5)	9 (5.7)	34 (21.4)	16 (10.1)	30 (18.9)	17 (10.7)	159
*p* value	0.118		0.549		< 0.001		0.019		
T_1_–T_2_	46 (27.0)		32 (14.1)		52 (34.9)		40 (27.6)		
	24 (14.1)	22 (12.9)	20 (8.9)	12 (5.2)	35 (22.4)	17 (12.5)	28 (18.2)	12 (9.4)	170
*p* value	0.824		0.057		< 0.001		0.004		
T_0_–T_2_	45 (23.4)		27 (14.1)		67 (34.9)		53 (27.6)		192
	25 (13.0)	20 (10.4)	17 (8.9)	10 (5.2)	43 (22.4)	24 (12.5)	35 (18.2)	18 (9.4)	
*p* value	0.405		0.039		< 0.001		0.001		

Values are expressed as number (percentage). Percentage of total remodeling that occurred at each time point in the anterior, superior, posterior, and lateral regions. The differences in frequency between medial and lateral parts of the anterior, superior, posterior, and lateral regions are indicated by the *p* value. *AL* anterolateral, *AM* anteromedial, *SL* superolateral, *SM* superomedial, *PM* posteromedial, *PL* posterolateral, *M* medial, *L* lateral, *T*_*0*_ initial timepoint, *T*_*1*_ middle timepoint; *T*_*2*_ final timepoint.

**Figure 4:**
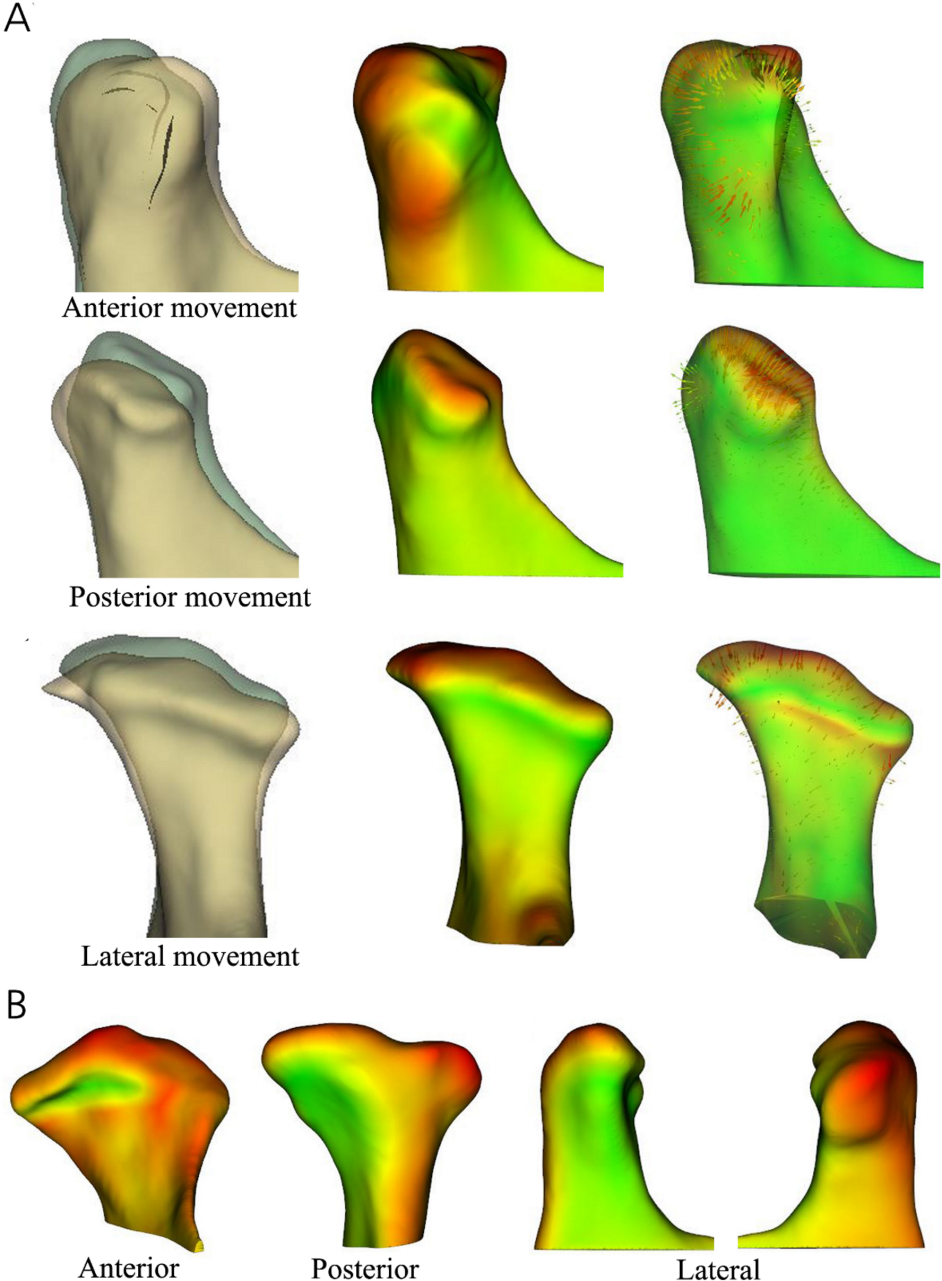
(**A**) Anterior, posterior, and lateral movements according to the condylar resorption and apposition. (**B**) Changes in resorption and apposition of medial and lateral parts of the condyle. Red and green colorations show bone resorption and apposition, respectively, 3D slicer (v. 4.11.0, Slicer, http://www.slicer.org).

The apposition and resorption patterns differed. In contrast to resorption, which was mainly observed in the superior region, apposition was more frequent in the posterior region. In the posterior, anterior, and lateral regions, apposition was observed at high rates in decreasing order in the posterior, lateral, and anterior regions (Table 2; Fig. 4A).

When the differences between the medial and lateral parts were compared, resorption occurred at a higher frequency in the lateral than in the medial part in the superior, posterior, and lateral regions. However, apposition was observed more frequently in the medial than in the lateral part in all of the regions. Particularly, the difference was statistically significant between the medial and lateral parts in the posterior and lateral regions where high apposition was observed (Table 2; Fig. 4B).

The changes over time for each condylar region suggest that, although resorption occurred the most in the superior region, the resorption rate and frequency did not continuously increase from T_0_ to T_1_ to T_2_ (T_0_–T_1_: 45.0%, T_1_–T_2_: 37.0%, T_0_–T_2_: 43.7%). However, the resorption frequency ultimately increased from T_0_ to T_2_. Such changes were also observed in the anterior, posterior, and lateral regions. Apposition was mainly observed in the posterior region; however, the frequency and rate were also not linear from T_0_ to T_1_ to T_2_ (Table 2).

## Discussion

In this study, we assessed the condylar resorption and apposition patterns in patients with TMJ osteoarthritis. Previous studies had short follow-up periods and limited observation ranges^7,20^. The studies were also limited as 3D features were not available, and the resorption patterns could not be quantified as only 2D assessment was used for the CBCT scans. However, Cevidanes^13^ reconstructed condyles in 3D and compared the condyles of long-term osteoarthritis patients to those of early osteoarthritis patients. The sites and resorption amounts were evaluated through comparison with a control group; however, changes over time could not be assessed. Moreover, changes in the individual condyles, assessed by normalizing the entire model to an average model, could not be evaluated. Osteoarthritic condylar resorption varies from patient to patient. Therefore, this study used CBCT scans to assess the changes in each patient’s individual condyles, and the long term changes through 5-year follow-up.

In previous studies, where large amounts of resorption and apposition led to condylar changes, the superimposition methods were inadequate. In previous studies, the anterior cranial base or voxel was overlaid, and an alternative technique of overlaying the mandibular 3D model with the condylar head as the reference was used^8,21,22^. However, significant condylar resorption leads to changes in the condylar position with respect to the cranial base. Thus, superimposition based on the anterior cranial base does not allow for the comparison of the amount or site of condylar resorption^21^. Overlay based on 3D models overcomes these limitations and allows for measurement of the morphology changes and resorption and apposition amounts without changing the condylar position. However, as the condylar size and shape changed over time, the condylar head based superimposition caused significant errors^10,18,22^. Voxel-based registration has the advantage of superior performance in general cases. However, in the case of patients with TMJ osteoarthritis, there is a problem that it is difficult to perform general voxel superimposition, or the accuracy is rather inferior when the change is severe in the condyle region. Even if voxel superimposition centered on the coronoid process is performed, since the coronoid region is not a large region, a slight error in the coronoid process may appear large in the condylar region. Above all, since the voxel superimposition is AI-based method that the program takes care of, it was not easy to correct the error that occurred afterwards. To correct this error, we used ROI surface registration, a method that uses both landmark registration and surface registration. Since ROI surface registration sets a region of interest including landmarks and surfaces on two 3D models, repetitive superimposition to reduce errors was possible by resetting the area. Also, the direction and amount of the error could be quantified by comparing the coordinates of the landmarks before and after. If the criterion was not satisfied, the ROI was finely adjusted, and superimposition was repeated until the criterion was satisfied. Through this process, it was possible to increase the accuracy of the superimposition.

Evaluation of the condylar changes during the average follow-up period of 5 years showed that osteoarthritis leads to both resorption and apposition. Moreover, although the frequency of resorption was higher, resorption was sometimes followed by apposition and vice versa (Fig. 2). In the superior region, where resorption was mainly observed, the frequency of resorption decreased from 86 to 74 and then increased to 97 from T_0_ to T_1_ to T_2_, respectively. These findings suggest that in TMJ osteoarthritis the condyle was continuously resorbed and, simultaneously, deposited and healed depending on the treatment time and effects. However, the resorption frequency increased from T_0_ to T_2_ as the resorption amounts and rates were greater than those of apposition.

Moreover, in patients with TMJ osteoarthritis, resorption and apposition patterns differed by condylar site. In every time period, the superior region mainly showed resorption. In contrast, the anterior, posterior, and lateral regions were observed with a higher frequency of apposition than resorption. In detail, comparison between the medial and lateral parts of the anterior, superior, posterior, and lateral regions showed that a higher frequency of resorption was observed in the lateral part than in the medial part. Conversely, a higher frequency of apposition was observed in the medial parts than in the lateral parts of the anterior, superior, posterior, and lateral regions. In particular, we observed significantly more apposition in the medial parts of the posterior and lateral regions. In other words, apposition occurred somewhere in the anterior, posterior, and lateral regions to compensate for the resorption force in the superior condylar region.

Comparison of apposition of the anterior, posterior, and lateral condylar regions showed that the apposition frequency was higher (in decreasing order) in the posterior, lateral, and anterior regions. This suggests that the condyle mainly moved (in decreasing order) in the posterior, lateral, and anterior regions to compensate for superior region resorption caused by osteoarthritis. Furthermore, resorption was the greatest in the superior region, and (in decreasing order) the posterior, lateral, and anterior condylar movements were more evident with time from T_0_ to T_1_ and T_2_. These findings suggest that the resorption and apposition patterns in the superior region, and posterior, lateral, and anterior regions, respectively, become more intense over time.

A study that compared average symptomatic and asymptomatic condylar models observed resorption at the lateral and medial poles of the anterior and posterior surfaces, respectively^12^. Resorption was observed in an average model of long-term osteoarthritis patients, except in the superior portion of the lateral pole^13^. This study demonstrated that resorption and apposition patterns are different for each patient and condyle. Although there were differences in the frequency, both resorption and apposition were observed in the superior, anterior, posterior, and lateral regions. Additionally, the shape and size varied for each condyle. Therefore, an average model cannot identify the various resorption patterns of individual condyles. Moreover, normalizing condyles of various sizes leads to inaccurate evaluation of the changes caused by osteoarthritis.

These changes lead to resorption of the superior part and, consequently, an open bite or asymmetry that causes changes in the skeletal relationship. After follow-up for 5 years, we observed that resorption was more dominant than apposition in the superior region that affects the skeletal relationship. Therefore, although the effect of splint therapy or medication on condylar bone resorption caused by osteoarthritis is controversial, they may be effective treatment methods if they can slow down bone resorption^21–24^.

The limitation of this study is that the numbers of condyles with apposition were less than those with resorption in the comparison of resorption and apposition. Such differences were attributed to the fact that we only included osteoarthritis patients in the study and the main change induced by osteoarthritis is condylar resorption^25^. Moreover, patients who did not receive treatment could not be included for ethical reasons. Therefore, additional studies comparing the resorption and apposition patterns by increasing the number of models with apposition and comparative studies between control and osteoarthritis models are required^26^. In addition, in this study, the amount of resorption and apposition was measured based on the closest point, but it can also be compared by matching the corresponding points before and after. For this, it is required to set the corresponding points before and after by projecting and matching the points to the sphere. In 3D slicer, this process is called Point Distributed Models (PDM) using Spherical Harmonic Representation (SPHARM-PDM). However, in this process, the model is mapped with a limited number of points and the shape of the model is simplified a lot. Especially in the case of TMJ OA, the shape of condyle is complex and the detailed shape is important. Therefore, comparing with this simplified shape can cause a big difference from the actual.

In this study, we assessed the condylar changes affected by osteoarthritis over a long period of time. By following-up for more than 5 years and adding T_1_ in addition to T_0_ and T_2_, we observed that condylar resorption and apposition not only occurred continuously but also simultaneously. Moreover, 3D modeling and superimposition methods that designated condylar areas showed that the condyle was not entirely resorbed, but that there are different areas where resorption or apposition mainly occurs. The findings suggested that resorption and apposition mainly occurred in the superior and posterior regions, respectively, leading to posterior condylar movement. Furthermore, condylar apposition occurred in the medial surface, close to the articular eminence in response to resorption in the superior surface. The finding that resorption was greater and faster than apposition led to the conclusion that treatments such as splinting may be effective if they can slow condylar resorption. Additionally, resorption and apposition of individual condyles can be quantified through 3D reconstruction and a new superimposition method. The 3D reconstruction and the new superimposition method used in this study may be beneficial for future TMJ studies.

TMJ osteoarthritis is a disease that requires special attention in dental treatment. When TMJ is deformed due to osteoarthritis, the occlusion gets much more changed, making treatment difficult. Also, osteoarthritis is a disease that changes over a long period of time. Therefore, understanding the long-term changes in the TMJ caused by osteoarthritis can be very important in actual clinical practice. Improved superimposition method in this study opened the way to accurately diagnose and treat condyles affected by osteoarthritis. The visualization of changing condyle through an overlaid model and color map added the depth and understanding of TMJ osteoarthritis. The pattern of changes in the TMJ can also help to establish a treatment strategy during treatment of the TMD or orthodontic treatment. Since 3D data on changes in TMJ osteoarthritis over a long period of time cannot be easily collected, the value of this study can be found.

## Methods

### Subjects

This retrospective study included 67 condyles of 43 patients (mean age: 28.2 years, standard deviation: 8.3) who visited or were referred to a dental clinic that specialized in orofacial pain with symptoms of temporomandibular joint disorders (Table 3). CBCT (DCT-90-P; 50-90KvP, 2–10 mA, 24 s, voxel size 0.2 mm, field of view 120 × 85 mm; VATECH Co., Ltd., Hwaseong-si, South Korea) records were collected for all patients. We seated the patients and asked them to rest their heads in the center of a proprietary headrest. They were then instructed to bite into the intercuspal position. We lined the center beam up with the sagittal plane and adjusted the seat position so that the lateral crossed cursor was targeted at the condyle. The scanned data were automatically exported in the Digital Imaging and Communications in Medicine (DICOM) format.

**Table 3:** Characteristics of the sample patients.

	N	Age (yr)	Treatment duration (T_2_–T_0_) (yr)
**Sex**			
Female	33	29.3 ± 9.4	6.2 ± 1.0
Male	10	26.6 ± 3.0	6.4 ± 0.6
**TMJ OA Condyle**			
Bilateral	48	30.2 ± 9.5	6.5 ± 1.1
Unilateral	19	26.8 ± 6.4	6.0 ± 0.7
Total	67	28.2 ± 8.3	6.3 ± 1.0

Values are expressed as mean ± standard deviation. *TMJ* temporomandibular joint, *OA* osteoarthritis, *yr* year.

CBCT was performed at least 5 years apart from T_0_ to T_2_. CBCT was performed again at T_1_ between T_0_ and T_2_. The mean follow-up period was 6 years and 3 months.

Patients were diagnosed with TMJ osteoarthritis based on medical history and clinical and radiological data according to Diagnostic Criteria for Temporomandibular Disorders (DC/TMD). As a diagnostic criterion for imaging, TMJ osteoarthritis can be diagnosed if there are destructive changes such as subchondral cyst, erosion, generalized sclerosis, or osteophyte on TMJ CT. This diagnosis was made by three oral medicine specialists with more than 10 years of experience. As the patients visited the clinic for clinical purposes, all patients were selectively administered physical, splint, and exercise therapies and medication. The splint was created using acrylic resin guided to a centric relation position.

Exclusion criteria were (1) systemic medical condition involving the immune system, degenerative musculoskeletal system, or neuropathy sequelae; (2) craniofacial syndromes; (3) facial trauma related to the TMJ; and (4) history of orthodontic treatment and orthognathic surgery. The study was conducted according to the guidelines of the Declaration of Helsinki, and approved by the appropriate Institutional Review Board of the Korea University Hospital (KUIRB-2019-0344-01). Informed consent was obtained from all participants or their legal guardians. All experiments were performed in accordance with relevant guidelines and regulations.

### Image preparation

#### 3D reconstruction

**Figure 5:**
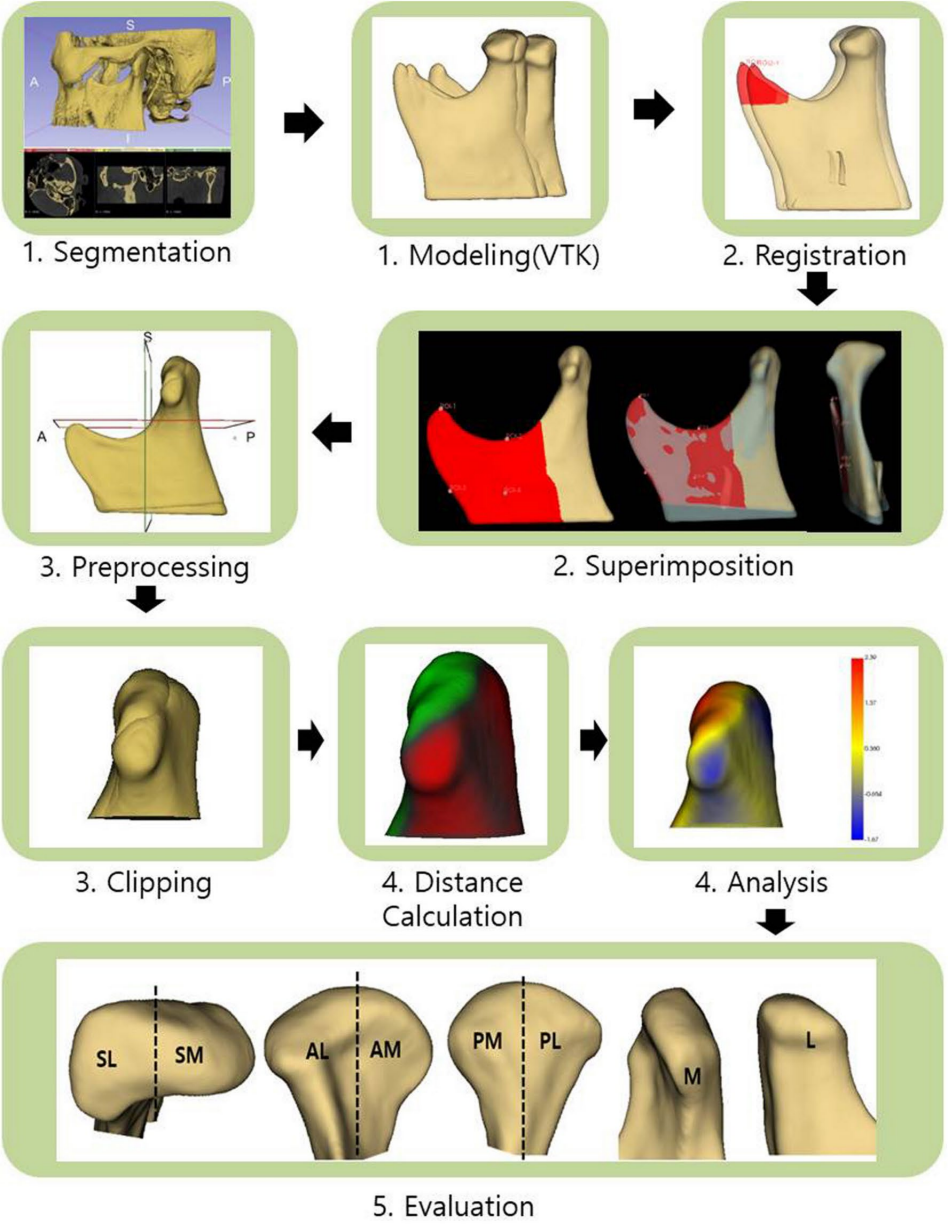
Technical route of the study. 1. Constructing the 3-dimensional mandibular segmentations. 2. Performing regional registration and superimposition based on the coronoid process, sigmoid notch and mandibular body 3. Generating the condylar surface model 4. Measuring the distance and color; vector map generation 5. Evaluating resorption and apposition after area specification, 3D slicer (v. 4.11.0, Slicer, http://www.slicer.org).

Semi-automatic discrimination procedures were performed to obtain 3D condylar models using DICOM files taken by CBCT. A 3D slicer (v. 4.11.0, Slicer, http://www.slicer.org) was used to set the condyle, mandibular body, and mandibular ramus as regions of interest (ROIs). The 3D model was constructed using discrimination procedures that outlined the mandibular ramus and condylar cortical boundaries, checked slice by slice on 3 planes, and allowed for manual editing. We segmented TMJ using the 3D slicer's segment editor module. The bone region was first extracted by adjusting the threshold and then segmentation was performed manually. For each coronal, axial, and sagittal slice, fine corrections were made using level tracing, paint, draw, and erase functions. This process was carried out by two observers and mutually verified. Then, the constructed 3D models were saved in the VTK file format (Fig. 5).

#### Model superimposition

To observe the osteoarthritic condylar changes, we superimposed the T_0_, T_1_, and T_2_ models of each patient. A stable reference was required for accurate superimposition. Thus, the mandibular body and coronoid process were used as a reference. We used the 3D slicer’s ROI surface superimposition, and the coronoid process (Cor), sigmoid notch (R3), and anterior point of mandibular ramus (R1) were designated as common areas for overlay of the T_0_, T_1_, and T_2_ models. The ROI of each designated area was expanded with the value of radius set to 60, including the coronoid process and the mandibular body. T_1_ and T_2_ models were then superimposed with the T_0_ model as a reference. After the superimposition, the overall accuracy of the coronoid process area was checked through the overlaid models and color map, and the distance between landmark points was measured to determine whether it was within 0.2 mm. If the criterion was not satisfied, the ROI was finely adjusted, and superimposition was repeated until the criterion was satisfied. The criterion of 0.2 mm was determined by judging when a satisfactory level of superimposition was achieved through the pilot study. This amount of error was judged as a level that could occur during the processes of superimposition. Through this process, it was possible to increase the accuracy of the superimposition.

Condylar changes over time could be observed more accurately based on the coronoid process and mandibular body. Moreover, by overlaying individual condyles, we could assess the osteoarthritic changes on each condyle (Fig. 5).

### Data analysis

#### Resorption and apposition amounts

**Figure 6:**
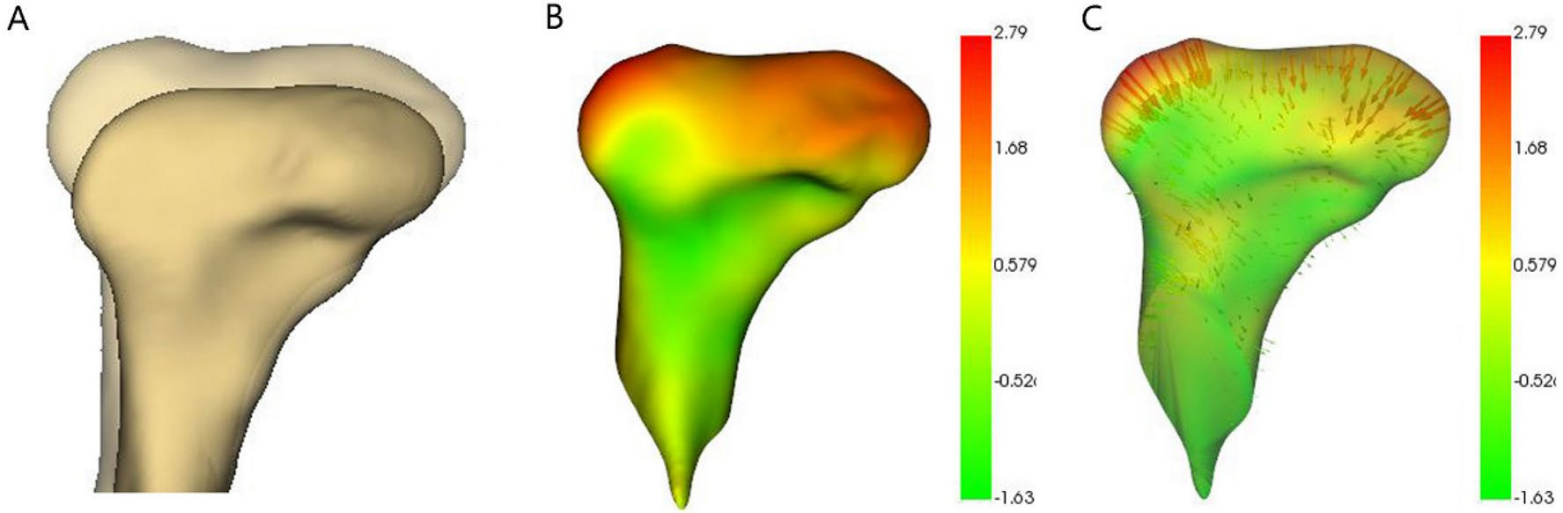
Evaluation methods. (**A**) Semi-transparent overlay. (**B**) Color map. (**C**) Vector map. Red and green colorations show bone resorption and apposition, respectively, 3D slicer (v. 4.11.0, Slicer, http://www.slicer.org).

Color coded magnitude and difference vector maps of the superimposed model in the 3D slicer were used to evaluate the direction and amount of condylar bone resorption and apposition based on the closest point. Then, the resorption and apposition amounts were quantified for each condyle by measuring the maximum corresponding surface distance. The mean and per year resorption and apposition amounts were calculated based on these data. In addition, differences between the sexes in resorption and apposition amounts were also calculated (Fig. 6).

#### Resorption and apposition sites

Condylar resorption and apposition were assessed by dividing the condyle into different areas to assess the resorption and apposition sites at T_0_, T_1_, and T_2_. First, the condyle was divided into superior, anterior, posterior, and lateral regions. Each was then subdivided into medial and lateral parts to observe whether resorption or apposition occurred in each region and subregion (Fig. 5). To observe changes over time, resorption and apposition were assessed at each region from T_0_ to T_1_, T_1_ to T_2_, and T_0_ to T_2_.

Color-coded and difference vector maps, and semi-transparent overlays were used on the T_0_–T_1_, T_1_–T_2_, and T_0_–T_2_ superimposed models to count the number of resorbed and apposed condylar sites (Fig. 6).

### Statistical analysis

All statistical analyses were performed using the Statistical Package for Social Sciences for Windows version 20.0 (IBM Corp., Armonk, NY, USA). Differences were considered significant at *p* < 0.05. Mean values of the maximum and average distances and standard deviations of each condyle were calculated, reflecting condylar surface changes.

The condylar remodeling types were evaluated by frequency. McNemar's test was performed to compare the condylar surface changes between the medial and lateral parts of each condylar region. A paired t-test was conducted to compare the condylar surface changes between the sexes.

## Data Availability

The data underlying this study will be available on reasonable request to the corresponding author.
